# Availability of Specific Direct Oral Anticoagulant Reversal Agents in US Hospitals

**DOI:** 10.1001/jamanetworkopen.2021.10079

**Published:** 2021-05-14

**Authors:** Zahir Kanjee, Marissa L. McCann, Jason A. Freed

**Affiliations:** 1 Division of General Medicine, Beth Israel Deaconess Medical Center, Boston, Massachusetts; 2 Department of Medicine, Harvard Medical School, Boston, Massachusetts; 3 Department of Pharmacy, Beth Israel Deaconess Medical Center, Boston, Massachusetts; 4 Division of Hematology and Hematologic Malignancies, Beth Israel Deaconess Medical Center, Boston, Massachusetts

## Abstract

This cross-sectional study assesses the availability of the direct oral anticoagulant reversal agents idarucizumab and andexanet alfa in US hospitals.

## Introduction

Direct oral anticoagulants (DOACs) are widely prescribed but associated with risk of major hemorrhage. Specific reversal agents have been developed for dabigatran (idarucizumab) and factor Xa inhibitors (andexanet alfa). Consensus statements from several^1,2,3^ but not all^4^ major organizations recommend these specific reversal agents, if available, over alternatives, such as prothrombin complex concentrates (PCC), in DOAC-associated life-threatening bleeding. Given the importance of timely reversal in life-threatening hemorrhage, we assessed the availability of specific DOAC reversal agents in US hospitals.

## Methods

This cross-sectional study followed the Strengthening the Reporting of Observational Studies in Epidemiology (STROBE) reporting guideline and was deemed non–human participant research and exempt from review by the Beth Israel Deaconess Medical Center Committee on Clinical Investigation. We used the Medicare Hospital Compare database to find eligible hospitals in the United States that provide emergency care, excluding those in territories, those operated by the Department of Defense, and those primarily designated as pediatric or psychiatric facilities. We searched each included hospital on pharmaceutical company drug locator websites for idarucizumab and andexanet alfa to assess whether they did or did not have access to each drug. Searches were conducted on manufacturer websites between March 11, 2020, and November 11, 2020, and analyzed from March 11, 2020, to February 15, 2021. Per the websites, the idarucizumab database was accurate as of May 31, 2019, and the andexanet alfa database was accurate as of March 31, 2020. Eligible hospitals were categorized as trauma centers if they were designated level 1 or 2 by the American Trauma Society. Information about our data sources can be found in the eMethods in the Supplement.

We provide descriptive statistics. We used χ^2^ tests to assess the association between hospital attributes (ie, hospital type, trauma status, state) and access to idarucizumab and, separately, andexanet alfa. All analyses were performed with Stata version 13 (StataCorp). Maps were completed using the spmap and maptile commands. Statistical significance was set at *P* < .05, and all tests were 2-tailed.

## Results

There were 5340 hospitals in the Medicare Compare database. Of these, 4276 (80.1%) met inclusion criteria (Table). Among these hospitals, 2562 (59.9%) had idarucizumab, and 499 (11.7%) had andexanet alfa. Availability of medications varied by hospital type for both idarucizumab (χ^2^ = 831.8; *P* < .001) and andexanet alfa (χ^2^ = 139.6; *P* < .001). Among 528 trauma centers, 503 (95.3%) had idarucizumab, and 151 (28.6%) had andexanet alfa. The proportion of hospitals with access to idarucizumab (range, 22%-100%; χ^2^ = 576.7; *P* < .001) and andexanet alfa (range, 0%-50%; χ^2^ = 249.9; *P* < .001) varied by state (Figure).

**Table.** Hospitals With Idarucizumab and Andexanet Alfa

Characteristic	Hospitals, No. (%)			
	Idarucizumab available		Andexanet alfa available	
	Yes	No	Yes	No
All hospitals (N = 4276)	2562 (59.9)	1714 (40.1)	499 (11.7)	3777 (88.3)
Hospital type				
Acute care (n = 2950)	2195 (74.4)	755 (25.6)	459 (15.6)	2491 (84.4)
Critical access (n = 1326)	367 (27.7)	959 (72.3)	40 (3.0)	1286 (97.0)
Trauma level status				
Not a trauma center (n = 3748)	2059 (54.9)	1689 (45.1)	348 (9.3)	3400 (90.7)
Trauma center				
Trauma level 1 or 2 (n = 528)	503 (95.3)	25 (4.7)	151 (28.6)	377 (71.4)
Trauma level 1 (n = 217)	204 (94.0)	13 (6.0)	79 (36.4)	138 (63.6)
Trauma level 2 (n = 311)	299 (96.1)	12 (3.9)	72 (23.2)	239 (76.8)

**Figure.**
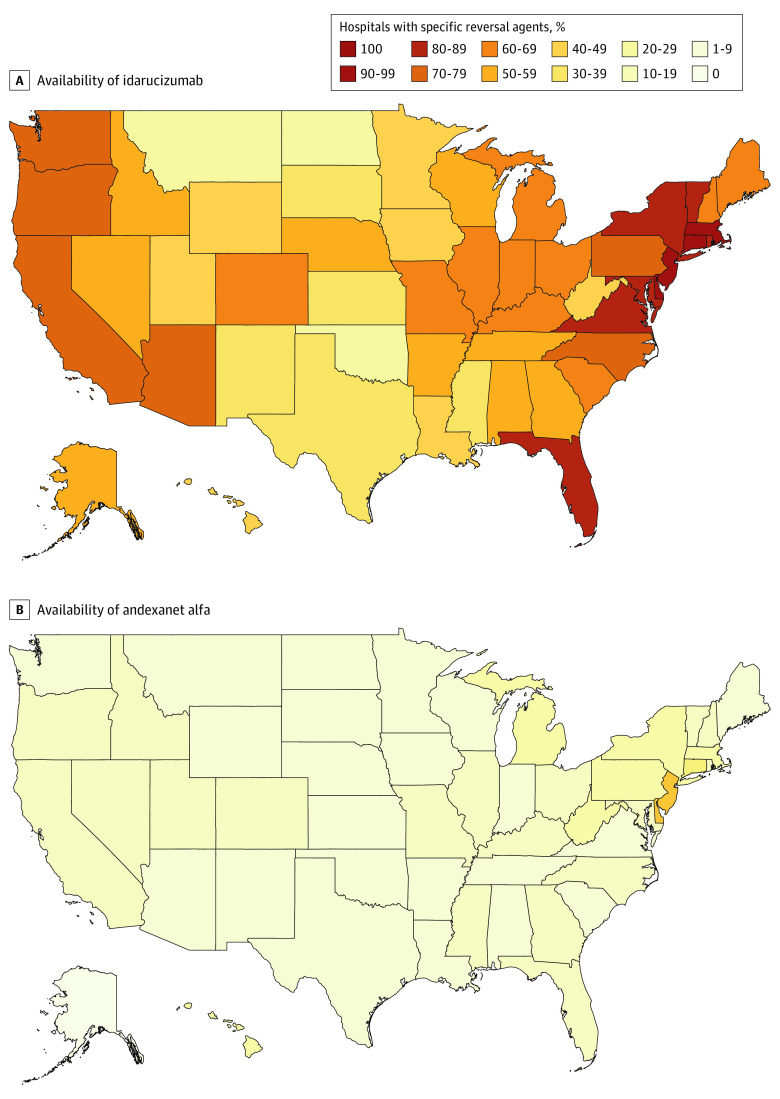
Reversal Agent Availability by State

## Discussion

In our study of 4276 US hospitals providing emergency care, we found a significant discrepancy in the availability of andexanet alfa and idarucizumab. Despite factor Xa inhibitors being prescribed for atrial fibrillation 20 times more frequently than dabigatran,^5^ acute care hospitals and trauma centers were 5 times and 3 times less likely, respectively, to have access to andexanet alfa than idarucizumab. The availability of each medication varied substantially across states and regions. To our knowledge, this is the first assessment of national availability of DOAC reversal agents.

Limitations of this study include the use of nonvalidated industry data related to medication purchases rather than confirmation of stock on hand, which could potentially overestimate availability if stock is exhausted and not replenished. Second, company data were slightly out of date and from different time periods, but we do not anticipate that these factors affected the overall results. Third, idarucizumab was approved by the US Food and Drug Administration in October 2015, while andexanet alfa was approved in May 2018. However, we saw negligible increases in andexanet alfa availability between December 2019 and March 2020 (data not shown), so we do not think this is a significant limitation. Fourth, we did not assess the availability of 4-factor PCC, which some hospitals may have chosen to stock rather than andexanet alfa.

## Notes

Supplement. **eMethods.** Data Sources eReferences.
