# Supplementary material for: Availability of Specific Direct Oral Anticoagulant Reversal Agents in US Hospitals
Source: JAMA Netw Open. 2021 May 14;4(5):e2110079. doi: 10.1001/jamanetworkopen.2021.10079 (PMC8122227; doi:10.1001/jamanetworkopen.2021.10079)
Supplement: Supplement. — eMethods. Data Sources eReferences. [file jamanetwopen-e2110079-s001.pdf]

Supplemental Online Content

Kanjee Z, McCann ML, Freed JA. Availability of specific direct oral anticoagulant reversal agents in US hospitals. *JAMA Netw Open.* 2021;4(5):e2110079.
doi:10.1001/jamanetworkopen.2021.10079

eMethods. Data Sources

eReferences.

© 2021 Kanjee Z et al. *JAMA Network Open.*

eMethods. Data Sources

We used the Medicare Hospital Compare database[1] to find eligible hospitals in the United States. Availability by hospital was determined by pharmaceutical company drug locator websites.[2,3] Trauma center designation was determined with the American Trauma Society Find Your Local Trauma Center tool.[4]

eReferences.

1. Centers for Medicare & Medicaid Services. Explore and download Medicare provider data. Accessed March 1, 2020. https://data.medicare.gov/widgets/xubh-q36u

2. Praxbind. Find praxbind. Accessed May 13, 2020. https://www.praxbind.com/find-praxbind

3. Find Hospitals That Use Andexxa. Andexxa. Accessed May 13, 2020. https://andexxa.com/locator/

4. American Trauma Society. Find your local trauma center. Accessed June 1, 2020. https://andexxa.com/locator/

© 2021 Kanjee Z et al. *JAMA Network Open.*
